# Effect of the natural arsenic gradient on the diversity and arsenic resistance of bacterial communities of the sediments of Camarones River (Atacama Desert, Chile)

**DOI:** 10.1371/journal.pone.0195080

**Published:** 2018-05-01

**Authors:** Carla G. Leon, Ruben Moraga, Cristian Valenzuela, Concetta Gugliandolo, Angelina Lo Giudice, Maria Papale, Claudia Vilo, Qunfeng Dong, Carlos T. Smith, Ramon Rossello-Mora, Jorge Yañez, Victor L. Campos

**Affiliations:** 1 Environmental Microbiology Laboratory, Department of Microbiology, Faculty of Biological Sciences, University of Concepción, Concepción, Chile; 2 Microbiology Laboratory, Faculty of Renewable Natural Resources, Arturo Prat University, Iquique, Chile; 3 Department of Chemical, Biological, Pharmaceutical and Environmental Sciences, University of Messina, Messina, Italy; 4 Institute for the Coastal Marine Environment, National Research Council (IAMC-CNR), Messina, Italy; 5 Center for Biomedical Informatics, Department of Public Health Sciences, Loyola University Chicago, Maywood, Illinois, United States of America; 6 Marine Microbiology Group, Institut Mediterrani d’Estudis Avancats (CSIC-UIB), Esporles, Spain; 7 Department of Analytical and Inorganic Chemistry, Faculty of Chemical Sciences, University of Concepcion, Concepcion, Chile; Wageningen University, NETHERLANDS

## Abstract

Arsenic (As), a highly toxic metalloid, naturally present in Camarones River (Atacama Desert, Chile) is a great health concern for the local population and authorities. In this study, the taxonomic and functional characterization of bacterial communities associated to metal-rich sediments from three sites of the river (sites M1, M2 and M3), showing different arsenic concentrations, were evaluated using a combination of approaches. Diversity of bacterial communities was evaluated by Illumina sequencing. Strains resistant to arsenic concentrations varying from 0.5 to 100 mM arsenite or arsenate were isolated and the presence of genes coding for enzymes involved in arsenic oxidation (*aio*) or reduction (*arsC*) investigated. Bacterial communities showed a moderate diversity which increased as arsenic concentrations decreased along the river. Sequences of the dominant taxonomic groups (abundances ≥1%) present in all three sites were affiliated to *Proteobacteria* (range 40.3–47.2%), *Firmicutes* (8.4–24.8%), *Acidobacteria* (10.4–17.1%), *Actinobacteria* (5.4–8.1%), *Chloroflexi* (3.9–7.5%), *Planctomycetes* (1.2–5.3%), *Gemmatimonadetes* (1.2–1.5%), and *Nitrospirae* (1.1–1.2%). Bacterial communities from sites M2 and M3 showed no significant differences in diversity between each other (p = 0.9753) but they were significantly more diverse than M1 (p<0.001 and p<0.001, respectively). Sequences affiliated with *Proteobacteria*, *Firmicutes*, *Acidobacteria*, *Chloroflexi* and *Actinobacteria* at M1 accounted for more than 89% of the total classified bacterial sequences present but these phyla were present in lesser proportions in M2 and M3 sites. Strains isolated from the sediment of sample M1, having the greatest arsenic concentration (498 mg kg^-1^), showed the largest percentages of arsenic oxidation and reduction. Genes *aio* were more frequently detected in isolates from M1 (54%), whereas *arsC* genes were present in almost all isolates from all three sediments, suggesting that bacterial communities play an important role in the arsenic biogeochemical cycle and detoxification of arsenical compounds. Overall, results provide further knowledge on the microbial diversity of arsenic contaminated fresh-water sediments.

## Introduction

Arsenic (As) is one of the most prevalent toxic metalloids on Earth, occurring primarily as the inorganic species oxyanion arsenate (H_3_AsO_4_) [As(V)] and arsenite (H_3_AsO_3_) [As(III)]. Arsenates are the predominant species in soil and oxygenated surface water, whereas arsenite predominates under anoxic or reduced conditions [1] and it is 100 times more toxic and 4 to 10 times more soluble than As(V) [2, 3]. Arsenic present in the environment originates from both natural and anthropogenic sources. There is also a growing concern of As contamination from agricultural and other anthropogenic sources, such as copper and sodium-based arsenicals from herbicides and pesticides [4].

Microorganisms in As-rich environments have evolved mechanisms to utilize arsenic for metabolic processes or to detoxify the cell. Therefore, they influence the biochemical cycle of As, bio-transforming As-species varying in solubility, mobility, bioavailability and toxicity [5–7]. While many heavy metals are essential elements at low concentrations, they can exert toxic effects at high concentrations, such as those present in polluted environments. In response to toxic concentrations of heavy metals, many aquatic organisms, including microorganisms, can develop tolerance. Some bacteria, representing different phylogenetic groups involved in As-transformation use processes such as reduction, oxidation and methylation mechanism to tolerate arsenic [8–11]. In general, two metabolic pathways are known for arsenate reduction, one serving for detoxification purposes while the other, respiratory pathway can be used for energy production. The detoxification pathway has been well studied and characterized. In brief, the *ars* system confers resistance to arsenic in prokaryotes, it is composed of up to 5 genes, *arsRDABC*, but at least three are required: *arsR*, encoding a transcriptional repressor, *arsB*, a transmembrane efflux pump, and *arsC*, an arsenate reductase. When present, the proteins encoded by *arsA* and *arsD* help the efflux pump encoded by *arsB* [12–15]. In the respiratory pathway, sometimes called dissimilatory arsenate respiration, a respiratory arsenate reductase consisting of two subunits (ArrA and ArrB) is responsible for the reduction of arsenate. The reductase is encoded by the *arr* operon, which always includes the *arrA* and *arrB* genes, with some strains containing an additional membrane subunit ArrC [13].

On the other hand, microbial arsenite oxidation is a well-known process and many microorganisms are able to do it by means of an arsenite oxidase protein, encoded by the *aio* system [16–21]. *aio* genes have been identified in bacteria isolated from various arsenic-rich environments [22]. Arsenite-oxidizing bacteria include chemolithoautotrophic and heterotrophic microorganisms, such as *Acidiphilium acidophilum*, *Acidithiobacillus ferrooxidans*, *Cenibacterium arsenoxidans*, *Alcaligenes faecalis*, *Cupriavidus necator*, *Hydrogenophaga* sp., *Sinorhizobium* sp., *Stenotrophomonas* sp. MM-7, *Ancylobacter dichloromethanicus* As3-1b [23–27] and some *Pseudomonas* spp. [28–30], as well as the thermophiles *Thermus aquaticus*, *Thermus thermophilus*, *Anoxybacillus flavithermus* TCC9-4 [31–33]. In addition, a novel arsenite oxidase gene, *arxA*, was identified in the genome of the Mono Lake (California, USA) isolate *Alkalilimnicola ehrlichii* MLHE-1, a chemolithoautotroph that couples arsenite oxidation to nitrate reduction [18].

Microorganisms mediate many important processes in the aquatic environment, including self-purification, nutrient recycling and development of heavy metal tolerance. The microbial community might even allow these important functions to be maintained despite the input of heavy metals into the environment [7, 9].

In northern Chile, As is leached out of volcanic materials naturally present in watershed areas of the Andes, resulting in high concentrations of As (0.1–1.5 mg L^-1^) in waters [27, 33–38], representing a serious health concern for populations using waters from Camarones river for both human consumption and agricultural activities. The role of microorganisms in the mobilization and speciation of arsenic has been studied, and several As-resistant bacteria from this river have been previously reported [27, 37–38].

In order to unveil the members of the bacterial community present in the arsenic rich sediments of the Loa river and the changes in these communities as consequence of a gradient of arsenic concentrations in this river, the taxonomic and functional characterization of bacterial communities associated with metal-rich sediments were evaluated using a combination of genomic approaches. Phylogenetic diversity of *Bacteria* has been investigated by the next generation sequencing technique (16S rRNA Illumina sequencing), a molecular approach allowing to simultaneously reveal a large number of individuals and their affiliation. Arsenic resistant isolates were characterized and investigated for the presence of genes coding for enzymes involved in the oxidation (*aio*) and reduction (*arsC*) of arsenic.

## Materials and methods

### Sample collection

**Fig 1:**
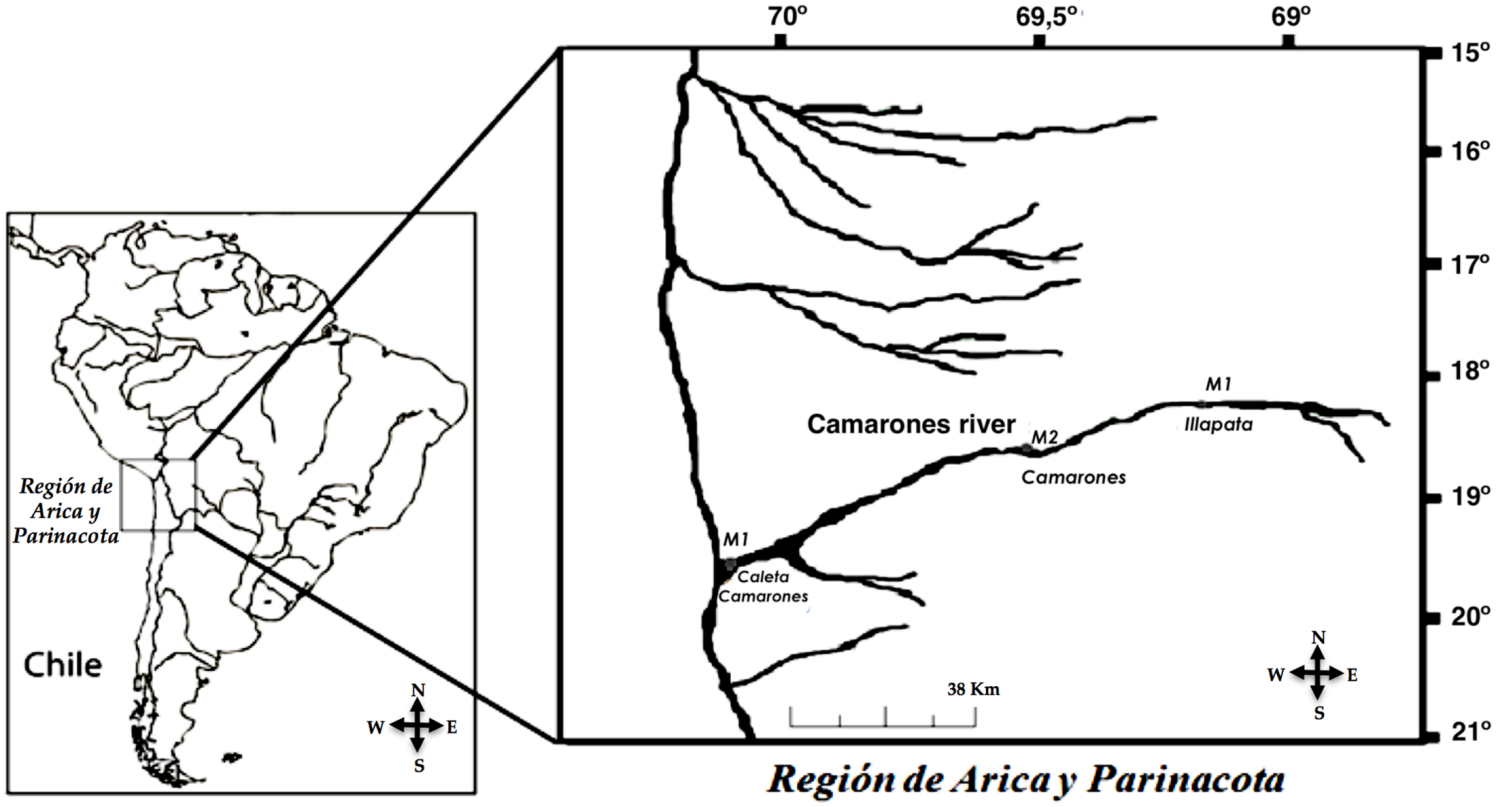
Map of the Chilean Arica y Parinacota region and location of M1, M2 and M3 sampling sites along the Camarones River.

According to article 589 of the Chilean Civil Code, no permissions are required to collect samples at the sites selected for this study. No endangered or protected species are present at the sampling places. Sediment samples were collected from three sites at Camarones river: the first (M1) is located close to Illapata, at the river upper course, where the flux of water is turbulent (18°56´53.88” S—69°30´45.64” W, 2,144 m above sea level); the second (M2) is located 38 km West of Illapata close to Camarones village (19°00´31.65” S—69°51´37.64” W, 709 m above sea level); the third (M3), located 47 km west M2, is close to Caleta Camarones at the mouth of the river (19°11´ 06.93” S—70°16´06.93” W, 13 m above sea level) (Fig 1).

Triplicate samples were obtained from the surface up to a depth of 10 cm using 15 cm long and 3 cm diameter cores. After collection, all samples were kept at 4°C during transport to the laboratory for further processing. After arriving at the laboratory, the superficial 2 cm (aerobic fraction) of each sediment sample were mechanically homogenized under sterile conditions in a laminar flow hood (ZHJH-C 1109C, Zhicheng, Korea).

### Physicochemical characterization of sediment samples

#### Environmental parameters

The pH and temperature were measured on superficial sediments, using the multi-parameter tester Hanna HI98195 (Hanna Instruments). Total organic matter (TOM) was determined gravimetrically following the weight loss by ignition technique [39]. This assay was done in triplicate and the results expressed as % of organic matter.

Total carbon (TC) was determined by combustion at 1300°C using a LECO CR-12 carbon analyzer. To measure total organic carbon (TOC), carbonates were eliminated acidifying the samples with HCl (1:1) and the remnant organic carbon was determined using a LECO CR-12 equipment. Inorganic carbon was determined as the difference between TC and TOC. Total nitrogen (TN) was determined using the micro-Kjeldhal technique modified by Branstreet [40]. Certified sediment standards, in accordance with the National Institute of Standards and Technology (NIST), were used for the calibration of carbon and nitrogen analyses. Carbon and nitrogen analyses were performed in duplicates and the results expressed as % in the sample.

#### Heavy metals concentration

Concentrations of copper (Cu), zinc (Zn), lead (Pb) and cadmium (Cd) were quantified using atomic absorption spectrophotometry, after microwave digestion of samples. Briefly, 0.5 g of sieved and dried sediment was added into 9 ml concentrated nitric acid *plus* 3 ml concentrated hydrochloric acid at 175°C for 10 min (US EPA 2007). After cooling down, the extracts were centrifuged at 3000 rpm for 5 min. Supernatant was analysed using an AA800 atomic absorption spectrophotometer (PerkinElmer).

Total As was determined in each sample using high performance liquid chromatography (HPLC). HPLC was coupled to a system of gaseous arsine formation and the As detection was achieved by atomic absorption in a quartz bucket (HPLC/HG/QAAS) [41].

### Prokaryotic cell abundance

Total prokaryotic cell counts (TC) were determined using 4′,6-diamidino-2-phenylindole (DAPI) staining (1 μg ml^−1^, final concentration). Triplicates of sediment suspensions (fixed with formalin at a final concentration of 2%) were sonicated three times for 1 min to detach cells from particles. Cells were collected on black polycarbonate membrane filters (0.2-μm pore size, 25 mm diameter, Nuclepore Corporation, Pleasanton, USA) and counted using epifluorescence microscopy (Motic BA310, at ×1000 magnification).

### Total bacterial community composition analysis

#### Genomic DNA extraction

The genomic DNA was extracted in triplicate directly from sediment samples (1 g) using PowerSoil DNA Isolation Kit (MO-BIO Laboratories, Inc., USA) according to the manufacturer’s direction. The extracts were subsequently purified. Quality and concentration of DNA were checked by UV/Vis spectroscopy (NanoDrop ND-1000, Peqlab, Erlangen, Germany). DNA was used as template for the high-throughput sequencing.

#### 16S rRNA gene massive sequencing (Illumina MiSeq)

The V1-V2 region of the 16S rRNA genes was amplified using the modified universal primers 27f (5'-AGAGTTTGATCCTGGCTCAG-3 ') and 338r (5'-GCTGCCTCCCGTAGGAGT-3'), at the “The Greehey Children’s Cancer Research Institute” (Greehey CCRI). The Illumina MiSeq Platform was used to generate V1-V2 amplicon reads in a paired-end sequencing run with a read length of 300 bp, at the UT Health Science Center in San Antonio (USA). All sequences were submitted to GenBank with the following numbers MG545618 to MG545646 (SUB3227168).

#### Analyses of bacterial communities

The raw data were analysed using the bioinformatics analysis software Mothur (version 1.35.1) with the default options, unless otherwise stated. Reads shorter than 200 bp were discarded. Reads were denoised using the “pre.cluster” command in Mothur platform to remove sequences that were likely due to errors and assemble reads which differed only by 2bp [42]. Chimeric sequences were identified and removed using UCHIME algorithm, and the remaining sequences classified against the SILVA database [43]. Alpha diversity measures (richness for observed species and Shannon diversity) were calculated on the OTU table obtained from all high-quality sequences. OTUs defined at 97% similarity level were selected and their relative abundance was normalized using the subsampling-based method described in Mothur (http://www.mothur.org/wiki/Normalize.shared) prior to comparative analyses. To compare the bacterial community compositions across groups of samples, Bray–Curtis similarity analyses were performed and similarity matrices were used to obtain CLUSTER graph, by using PRIMER 6.1.18 (Primer-E, Ltd).

### As-resistant strains

#### Bacterial isolation and tolerance levels to arsenic

For the isolation of As-tolerant bacteria, homogenized sediment (1 g) was added to a sterile solution of 10 mL 0.85% NaCl and agitated at 100 rpm for 5 min. Serial dilutions (0.1 mL aliquots) were cultured on R2A supplemented with 0.5 mM As(III) (as sodium arsenite) or 0.5 mM As(V) (as sodium arsenate) and incubated during 48 h at 25°C [44]. Colonies were randomly isolated from As-amended R2A and then screened for As-tolerance, as follows. As-tolerance of each isolate was determined using the agar dilution technique on plates of Luria Bertani (LB) agar containing from 0.5 to 100 mM sodium arsenite or sodium arsenate. Plates were inoculated with approximately 3×10^7^ CFU mL^-1^ cells obtained from each As tolerant strain and cultured at 25°C for 24 h. Agar plates containing bacteria but no metalloid were used as controls [45]. According to Rokbani et al. [46], isolates able to grow in the presence of at least 7 mM As (III) (arsenite) or 20 mM As (V) (arsenate) were considered as resistant.

#### As oxidation and reduction rate

The strains were grown in chemically defined medium (CDM) [44] supplemented with 0.5 mM arsenite or arsenate. Cultures were incubated at 30°C for 48 h and samples taken every 12 h to monitor As-conversion and bacterial growth (measured by absorbance at 600 nm). Oxidation of As(III) to As(V) and reduction of As(V) to As(III) were determined from supernatants of cultures filtered using sterile 0.22 μm filters (Millipore). As(III) and As(V) were measured by HPLC/HG/QAAS [41]. Samples (500 μL) were introduced to the HG system by means of an automatic injection valve. Samples were transported to the T-joint manifold using 10% hydrochloric acid as carrier and there continuously mixed with a solution of sodium borohydride to obtain AsH_3_. Gaseous arsine was separated from the liquid waste in a gas–liquid separator and carried, by a continuous flow of argon, to the atomizer. Atomic absorption signals were processed using commercial AA Winlab software (PerkinElmer). Quantification limit was 0.5 μg L^**−**1^ with a linear response up to 20 μg L^**−**1^ [28, 47].

#### Identification of As-resistant strains

Genomic DNA from isolates was obtained using UltraClean Microbial DNA Isolation Kit, following the manufacturer’s instructions (Mobio Laboratories, Inc.). The 16S rRNA genes were amplified by PCR using universal bacterial primers GM3 and GM4 (AGAG TTTG ATCM TGGC and TACC TTGT TACG ACTT, respectively) according to Brito-Echeverría et al. [48]. Amplified fragments were sequenced using the Dyenamic ET terminator kit (General Electric), in a 3100 Avant genetic Analyser (Applied Biosystem), following the manufacturer's instructions. A nucleotide BLAST search (http://www.ncbi.nlm.nih.gov/BLAST) was performed to obtain sequences with the greatest significant alignment.

#### Detection of detoxifying arsenate reductase *arsC* gene

To detect the *arsC* gene, isolates were cultured for 12 to 18 h at 25°C in LB supplemented with 0.5 mM NaH_2_AsO_5_ until reaching 10^8^ CFU mL^-1^. The genomic DNA of each isolate was extracted using the UltraClean Microbial DNA Isolation Kit (Mobio Laboratories, Inc.) to serve as template for PCR amplification. Primers used for the PCR technique were arsC-1-F (5' GTAATACGCTGGAGATGATCCG 3’) and arsC-1-R (5’ TTTTCCTGCTTCATCAACGAC 3'), which correspond to the *ars* operon of *Escherichia coli* [49]; and arsC-1-F (5' AGTCCTGTTCATGTGYAC 3’) and arsC-1-R (5' TGGCGTSGAAYGCCG 3') described for the *ars* operon of *Pseudomonas aeruginosa* and *Pseudomonas putida* [10, 49]. PCR products were separated by electrophoresis in a 1.2% agarose gel and visualized in an UV transilluminator after staining with ethidium bromide [23].

#### Detection of arsenite oxidation *aio* gene

Strains were cultured in LB supplemented with 0.5 mM NaH_2_AsO_3_, and DNA was extracted as described above. PCR was performed using the primers 69F (TGYA TYGT NGGN TGYG GNTA YMA) and 1374R (TANC CYTC YTGR TGNC CNCC) according to Valenzuela et al. [37]. *E*. *coli* S17-1 was used as positive control [50], while *E*. *coli* K-12 was used as negative control. Separation of amplified products was achieved by 0.8% agarose gel electrophoresis followed by ethidium bromide (0.5 μg mL^-1^) staining and the bands were observed on an UV transilluminator. The QIA quick PCR purification kit (Qiagen), following the manufacturer’s instructions, was used to purify the products obtained by PCR and these PCR products were sequencing by Macrogen (Korea). A homology search of 16S rRNA gene sequences was performed using the NCBI Basic Local Alignments Search Tool (BLAST) using the algorithm of the Nucleotide Blast program.

### Diversity indices and statistical analysis

It was replaced by “The experimental data was recorded using three replicates (n = 3) and data expressed as mean ± SD. Data was analysed using two-way ANOVA and Student’s T test using the GraphPad Prism 5 software. P values <0.05 were considered as statistically significant [51]. Associations between variables were calculated by Pearson’s correlation. Diversity indices and Fisher’s exact test was carried out in R software version 3.1.0. using the ‘diverse’ and ‘RVAideMemoire’ packages, respectively [52].

## Results

### Physical and chemical parameters

The pH, TOM, TOC, TN, As and others heavy metals (Cu, Zn, Pb and Cd) measured at each sampling site (M1, M2 and M3) along the Camarones river are reported in Table 1. Total organic matter (TOM), total organic carbon (TOC) and total nitrogen (TN) values were increased (ANOVA, p<0.001) at site M3 (>2.5%, >0.8% and >0.1%, respectively) and were significantly higher than those of M1 (p<0.001) and M2 (p<0.001) sites.

**Table 1:** Physical and chemical properties, metal composition and total prokaryotic cells (TC) in M1, M2 and M3 samples collected from Camarones River. TOM:total organic matter; TOC: total organic carbon; TN: total nitrogen; As: arsenic; Cu; copper; Cd: cadmium; Zn: zinc; Pb: lead.

	Physical-chemical properties				Metal composition					TC
Site	pH	TOM (%)	TOC (%)	TN (%)	As (mg/kg)	Cu (mg/kg)	Cd (mg/kg)	Zn (mg/kg)	Pb (mg/kg)	(cells g^-1^ × 10^8^)
M1	7.18±0.3	2.1	0.6	0.08	498±0.9	56.6±0.4	1.27±0.03	167.9 ± 1.7	47.6±4.2	8.1±1.0
M2	7.44±0.2	2.4	0.7	0.09	245±0.7	37.7±0.5	1.13±0.01	136.5±1.6	45.1±2.7	8.9±2.1
M3	7.49±0.1	3.9	1.3	0.14	128±0.6	45.0±0.4	0.82±0.14	87.6±1.9	32.6±3.2	8.8±1.2

The total As concentration in the sediment samples, measured by HPLC/HG/QAAS, decreased as the Camarones river approached the Pacific Ocean. Total As was 498 mg kg^-1^ at the Camarones rhithron zone (M1), it was 245 mg kg^-1^ at the medial zone (M2), and 128 mg kg^-1^ at the mouth of the river (M3) (Table 1). Total As at M1 site was significantly higher than M2 site (p = 0.0001) and M3 site (p<0.001), whereas M2 and M3 sites not presented significantly differences (p = 0.1038) (Table 1).

### Prokaryotic cell abundance

The abundance of total prokaryotic cells (TC), measured after DAPI staining, was similar at the three sites, ranging from 8.1 (M1) to 8.9×10^8^ cells g^-1^ (M2) (Table 1).

### Total bacterial community composition analysis

#### Sequencing data and diversity estimates

The Illumina-based analysis of the universal V1-V2 region of the 16S rRNA genes for Bacteria produced a total of 3,051,116 sequences across all samples. After quality check within the SILVA database and removing chimeras, 3,032,898 (99.4%) high quality sequences remained.

M1 showed the highest number of quality reads (1,163,206), and the highest number of OTUs was retrieved from sample M3 (26,512) (Table 2). M2 and M3 showed the highest’s Shannon diversity indexes. Non-parametric Chao1 and ACE estimators predicted that the highest richness was in M3, whereas the lowest was in M2.

**Table 2:** Sequencing information, diversity index (H’), estimator of richness (Chao1 and ACE) obtained by Ilumina sequencing from sediment samples (M1, M2 and M3) collected from Camarones River. *OTUs*: operation taxonomic units.

	M1	M2	M3
Number of reads	1,170,450	767,028	1,113,638
Number of high quality reads	1,163,206	763,126	1,106,566
Unique reads	138,797	127,315	157,358
% Unique reads	11.93	16.68	14.22
Shannon (*H*´)	3.82	6.13	6.10
OTUs at 97% genetic similarity	20,742	22,350	26,512
Chao 1	184,996	146,951	197,054
ACE	183,660	142,632	196,356

### Bacterial diversity

Retrieved OTUs were classified in a total of 31 different bacterial phyla, of which 26 were common to all samples. Sediment at the upper river site (M1) was, at phylum level, less rich (27 groups) than the other two sites, since four taxa were absent at M1 (*Deferribacteres*, *Elusimicrobia*, *Thermotogae* and SR1) (S1 Table).

**Fig 2:**
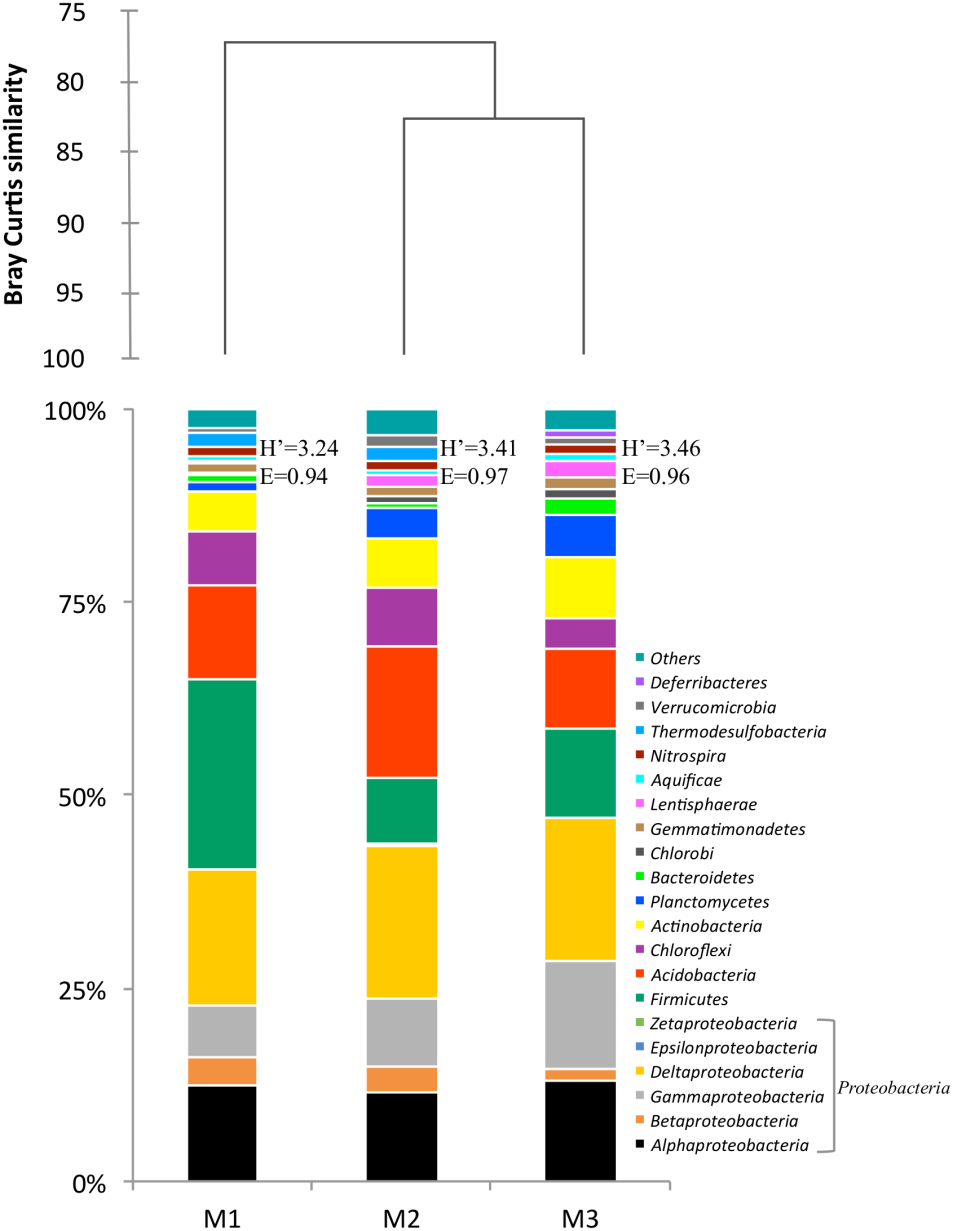
Cluster diagram and relative abundance of sequences (percentage) assigned to bacterial phylogenetic groups and proteobacterial subclasses from sediment samples (M1, M2 and M3) collected from Camarones River. *H’*: Shannon diversity index, *E*: Evenness index.

Overall, sequences of the dominant taxonomic groups (abundances ≥1%) across all sediment samples were affiliated with *Proteobacteria* (range 40.3–47.2% of total bacterial sequences), *Firmicutes* (8.4–24.8%), *Acidobacteria* (10.4–17.1%), *Actinobacteria* (5.4–8.1%), *Chloroflexi* (3.9–7.5%), *Planctomycetes* (1.2–5.3%), *Gemmatimonadetes* (1.2–1.5%), and *Nitrospirae* (1.1–1.2%). However, the relative abundance at phylum level varied considerably across samples, and changes in abundance of phylotypes determined different bacterial assemblages (Fig 2).

The composition of the bacterial communities of the different sediments varied because sequences affiliated to *Proteobacteria* (40.3%), *Firmicutes* (24.82%) and *Acidobacteria* (12.02%) accounted for more than 77% of identified bacterial sequences in M1. On the other hand, *Acidobacteria* (17.12%) were predominant in M2 while *Bacteroidetes* (2.09%), *Chlorobi* (3.94%), *Aquificae* (1.09%) and *Deferribacteres* (1.02%) were the dominant groups in M3, representing only minor components in M1 and M2.

Although *Deltaproteobacteria* (range 17.6–20.0%) represented the most abundant proteobacterial class, differences in relative abundance were also observed for sequences affiliated to the other classes: *Alphaproteobacteria* (12.41%-11.57%) was the second most abundant subclass in M1 and M2, while *Gammaproteobacteria* (13.91%) was the second most abundant subclass in M3. Sequences referred to *Epsilonproteobacteria* and *Zetaproteobacteria* represented minor components in all samples.

Relatively high abundant phyla in M1 and M2 included also *Thermodesulfobacteria* (1.64% and 1.89, respectively), but it constituted a minor component in M3 (0.18%). *Lentisphaerae* was dominant in M2 and M3, but not in M1, and finally *Verrucomicrobia* (1.32%) was dominant only in M2.

Among phyla whose abundance was ≤1%, sequences affiliated to BRC1, *Armatimonadetes*, *Cyanobacteria*/Chloroplast, *Fusobacteria*, *Deinococcus-Thermus*, *Caldiserica*, *Spirochaetes*, *Chlamydiae*, OD1, *Synergistetes*, TM7 and WS3 occurred across all sediments, even if at different relative abundances. Sequences related to *Tenericutes* were retrieved in M1 and M3, whereas those of *Eleusina* and *Thermotogae* were recovered in M2 and M3, but not in M1. Sequences affiliated with SR1 were found only in M3 (S1 Table).

A cluster diagram, representing similarities in the bacterial community composition (at phyla and proteobacterial classes level) of the studied sediments was done. This diagram showed that bacterial communities present at M2 and M3 sites grouped together, whereas the bacterial community at M1 site did not clustered with them (Fig 2).

From all samples, 934 genera were retrieved. The highest number of genera was observed in M3 (716), when compared to M2 (646) and M1 (436). The dominant bacterial genera (40), present in ≥ 1% of the total bacterial sequences at least in one of the three samples, are shown in Table 3. Richness at genus level slightly increased from M1 to M3 sites. A total number of 20 dominant genera were retrieved for M1, 19 for M2, and 25 for M3. A minor part of dominant bacterial genera (8/40) was ubiquitous in all samples: *Anaeromyxobacter*, *Desulfobacca*, *Hippea* (within *Deltaproteobacteria*), *Bacillus* (*Firmicutes*), Gp6 (*Acidobacteria*), *Sphaerobacter* (*Chloroflexi*), *Gemmatimonas* (*Gemmatimonadetes*) and *Nitrospira* (*Nitrospirae*). However, different abundant genera were unique for each site.

**Table 3:** Bacterial genera retrieved in sediment samples (M1, M2 and M3) from Camarones River.

Phylum	Class	Genus	M1	M2	M3		
*Proteobacteria*	*Alpha-*	*Afifella*					
		*Bauldia*					
		*Dongia*					
		*Pelagibius*					
	*Gamma-*	*Beggiatoa*					≥**8%**
		*Ectothiorhodosinus*					
		*Methylohalomonas*					≥**5%**
		*Thiococcus*					
	*Delta-*	*Anaeromyxobacter*					≥**3%**
		*Byssovorax*					
		*Desulfarculus*					≥**2%**
		*Desulfobacca*					
		*Desulfocurvus*					≥**1%**
		*Desulfovirga*					
		*Geothermobacter*					**<1%**
		*Hippea*					
		*Smithella*					
		*Sorangium*					
		*Syntrophorhabdus*					
*Firmicutes*		*Bacillus*					
		*Paenibacillus*					
		*Tumebacillus*					
		*Vulcanibacillus*					
*Acidobacteria*		Gp2					
		Gp3					
		Gp4					
		Gp6					
		Gp10					
		Gp21					
		Gp22					
*Chloroflexi*		*Caldilinea*					
		*Sphaerobacter*					
*Actinobacteria*		*Actinotalea*					
*Planctomycetes*		*Phycisphaera*					
		*Singulisphaera*					
*Chlorobi*		*Ignavibacterium*					
*Gemmatimonadetes*		*Gemmatimonas*					
*Lentisphaerae*		*Victivallis*					
*Nitrospirae*		*Nitrospira*					
*Thermodesulfobacteria*		*Caldimicrobium*					

The most abundant genus in M1 was *Bacillus*, whereas *Paenibacillus*, *Tumebacillus* and *Vulcanibacillus* were unique genera within *Firmicutes* for M1. Moreover, *Byssovorax*, *Desulfovirga* and *Sorangium* (*Deltaproteobacteria*), and Gp3 (*Acidobacteria*) were retrieved as unique dominant genera in M1. Four dominant genera were unique in M2: *Beggiatoa* (*Gammaproteobacteria*), *Geothermobacter* and *Smithella* (*Deltaproteobacteria*), and Gp2 (*Acidobacteria*), which also was the most abundant genus in M2.

Most of the dominant genera identified in M3 (13/40) if present accounted for less than 1% in the other samples, and were related to *Alphaproteobacteria* (*Pelagibius*, *Afifella* and *Bauldia*), *Gammaproteobac*teria (*Ectothiorhodosinus*, *Methylohalomonas* and *Thiococcus*), *Deltaproteobacteria* (*Desulfarculus*, *Desulfocurvus* and *Syntrophorhabdus)*, *Acidobacteria* (Gp 21 and Gp22), *Chlorobi* (*Ignavibacterium*), and *Actinobacteria* (*Actinotalea*).

The dominant genera common to M1 and M2 were related to *Dongia* (*Alphaproteobacteria*), Gp4 (*Acidobacteria*), *Caldilinea* (*Chloroflexi*) and *Caldimicrobium* (*Thermodesulfobacteria*). Genus Gp10 (*Acidobacteria*) was the only genus common to M1 and M3. Finally, dominant genera affiliated with *Planctomycetes* and *Lentisphaerae* were retrieved in M2 and M3.

### Arsenic-resistant isolates

#### Strains isolation and identification

Based on their ability to grow at increasing concentrations of arsenic, from 0.5mM to 100mM of As(III) or As(V), a total of 150 bacterial strains were isolated from M1, M2 and M3 sediments. As-resistant strains were defined as able to grow on agar containing 7 mM As(III) or 20 mM As(V) [52]. Of the total bacterial strains, 13 As-resistant strains were obtained from M1, 15 from M2, and 23 from M3 (Table 4). Overall, the 51 strains were capable to grow at concentrations of arsenite greater than 7mM (from 8 to 64 mM). Additionally, all the strains were capable of growth at the maximum concentration of arsenate tested (100 mM).

**Table 4:** As-resistant strains isolated from M1, M2 and M3 samples collected from Camarones River.

Strain	As^3+^ (*)	As^5+^ (**)	*arsC* (#)	*aio* (€)	O/R (&)	Strain	As^3+^ (*)	As^5+^ (**)	*arsC* (#)	*aio* (€)	O/R (&)
**Sediment M1**						**VC-65**	>40	>100	+	+	79/64
**VC-02**	40	>100	+	-	0/80	**VC-68**	10	>100	+	-	0/68
**VC-05**	10	>100	+	-	0/70	**VC-71**	>40	>100	+	+	84/65
**VC-07**	40	>100	+	+	90/95	**Sediment M3**					
**VC-08**	10	>100	+	-	0/75	**VC-72**	10	>100	+	-	0/25
**VC-11**	10	>100	+	-	0/98	**VC-73**	10	>100	+	-	0/36
**VC-17**	>40	>100	-	+	100/0	**VC-74**	10	>100	+	-	0/40
**VC-19**	>40	>100	+	+	96/95	**VC-77**	10	>100	+	-	0/32
**VC-21**	>40	>100	+	+	95/0	**VC-79**	10	>100	+	-	0/28
**VC-23**	>40	>100	+	+	93/75	**VC-81**	>40	>100	+	+	80/25
**VC-29**	10	>100	+	-	0/65	**VC-86**	10	>100	+	-	0/15
**VC-33**	>40	>100	+	-	0/70	**VC-87**	>40	>100	+	+	93/0
**VC-39**	>40	>100	+	+	95/55	**VC-88**	10	>100	+	-	0/25
**VC-41**	>40	>100	+	+	90/70	**VC-89**	>40	>100	+	-	0/30
**Sediment M2**						**VC-90**	10	>100	+	-	0/11
**VC-42**	10	>100	+	-	0/58	**VC-95**	>40	>100	+	+	87/10
**VC-44**	10	>100	+	-	0/55	**VC-97**	>40	>100	+	+	85/0
**VC-45**	>40	>100	+	-	0/60	**VC-102**	>40	>100	+	-	0/20
**VC-48**	>40	>100	+	+	75/63	**VC-114**	>40	>100	+	-	0/28
**VC-50**	>40	>100	+	-	0/53	**VC-119**	>40	>100	+	+	91/0
**VC-51**	10	>100	+	-	0/67	**VC-123**	>40	>100	+	+	85/20
**VC-52**	10	>100	+	-	0/64	**VC-131**	10	>100	+	-	0/20
**VC-53**	10	>100	+	-	0/60	**VC-139**	10	>100	+	-	0/22
**VC-56**	>40	>100	+	+	68/65	**VC-141**	10	>100	+	-	0/28
**VC-57**	10	>100	+	-	0/68	**VC-143**	10	>100	+	-	0/13
**VC-59**	10	>100	+	-	0/63	**VC-145**	10	>100	+	-	0/30
**VC-61**	10	>100	+	-	0/60	**VC-146**	10	>100	+	-	0/10

(*) Tolerance levels to As^3+^ expressed in mM of arsenite;

(**) Tolerance levels to As^5+^ expressed in mM of arsenate;

^(#)^ PCR detection *aox* gen;

^(€)^ PCR detection *arsC* gen;

^(&)^ Percentage of As^3+^ Oxidized or As^5+^ Reduced after 48 h of incubation at 37°C in the presence of As^3+^ or As^5+^, respectively.

Seventeen out of 51 As-resistant strains (33.3%) were able to oxidize As(III), 47 strains (90.2%) were able to reduce As(V), and 10 strains (19.6%) were able to both oxidize and reduce arsenic. All the 51 As-resistant strains were able to grow at the highest As(V) tested concentration (100mM) and 22 strains (43%) were able to grow up to 64 mM As(III).

The percentage of As(III)-oxidation was highest (90–100%) in strains from M1, ranged from 68 to 84% in strains from M2, and varied from 80% to 93% in strains from M3 (Table 4). The highest percentage of As(V) reduction was observed in strains from M1 (55–95%), and the lowest percentage in strains from M3 (10–40%) (Table 4). Pearson’s analysis showed a significant positive correlation (r = 1) between total As concentration and the capacity of strains from each sediment to oxidize or reduce the metalloid.

The levels of 16S rRNA sequence identity (99.1–100%) of As-resistant strains isolated from Camarones river to the most closely related species are reported in S2 Table. As-resistant strains were mainly identified as members of *Proteobacteria* (49 strains), including *Alpha-*(4 strains), *Beta-*(8 strains) and *Gammaproteobacteria* (37 strains), and *Firmicutes* (2 strains) (S2 Table).

#### Detection of *aio* and *arsC* genes

The presence of genes codifying for the enzymes arsenite oxidase (*aio*) and arsenate reductase (*arsC*) was investigated in all 51 As-resistant strains, *via* PCR. As(III)-oxidizing strains (17/51) presented a PCR product of approximately 1,200 bp, as expected size fragment corresponding to arsenite oxidase genes. The *aio* gene was more frequently detected in isolates from site M1 (41.2%) than from those of M2 (23.5%) or M3 (35.3%) (Table 4). In addition, As(V)-reducing strains (50/51) presented different PCR products, in relation to the presence of different arsenate reductases. The *arsC* gene was detected in all As-resistant strains, with the only exception of strain VC-17 from M1.

## Discussion

The northern Region of Chile, especially the Atacama Desert area, has been described as a naturally arsenic-rich environment. Minerals of metallic sulphides containing arsenic are dissolved in the Andes Mountains, affecting superficial and ground waters that cross the Atacama Desert which are used as drinking water sources. Since 1970, drinking water is treated to remove arsenic in all the large cities of the Atacama Desert, such as the city of Antofagasta. However, inhabitants of several small rural villages remain still exposed to arsenic in drinking water.

The inhabitants of the towns of Camarones and Illapata (Atacama Desert, Chile) use mainly natural water from the Camarones river for both human consumption and agricultural activities. Waters of Camarones river contain a total As concentration exceeding 1.0 mg L^-1^, mainly in the form of arsenate (As [V]). This contamination has chronically affected the rural populations living near the river, generating a variety of health problems [33, 53].

In this study, sediments (M1, M2 and M3) collected from Camarones river showed high total arsenic concentration which decreased (from 498 to 128 mg kg^-1^) as the river approached to the Pacific Ocean, since several inputs of springs and groundwater lacking As input into the lower zone of river (M3) [41]. Other authors reported similar total arsenic levels for this same river [33, 41]. Low values of total organic matter (TOM) were measured in M1 and M2 and may be due to the slopes and fast-flowing pattern of the Camarones river at these sites, dragging the organic matter. M1 and M2 showed also the highest concentrations of heavy metals Cu, Cd, Zn and Pb, attributed to the lithogenic characteristics of the river.

Metal-contaminated sediments are inhabited by extremely complex and well-adapted microbial communities, which play a fundamental role in degrading organic matter and in biogeochemical cycles [54–56]. Microbial diversity, here investigated by a massive parallel sequencing (Illumina), revealed a great diversity of bacterial communities, detecting also bacteria occurring at very low abundance (≤0.01%), that would have been masked by dominant populations if techniques with lower resolution had been applied. As evaluated by diversity indices, bacterial communities associated with sediments from the Camarones River possessed moderate diversity that increased along the course of the river from east (M1) to west (M3). The increase in bacterial diversity in sediment samples was associated with an increase in TOC and a decrease in As concentration. As generally accepted, moderately disturbed conditions, as lowering arsenic concentration on groundwater and sediments sample, often result in higher diversity of microbial communities [54–56], like those occurring at the mouth of Camarones river (site M3). Although adaptation of a community can take a long time (years), microorganisms present in metal contaminated soils may be selected in short periods of time (weeks or months) and after a period of time, microorganisms autochthonous of contaminated areas will be adapted to predominant conditions [57–58].

Sequences of the dominant taxonomic groups (abundances ≥1%) across all sediment samples were affiliated to *Proteobacteria* (mainly represented by *Delta-*, *Alfa-* and *Gamma-proteobacteria*) followed by *Firmicutes*, *Acidobacteria*, *Actinobacteria*, *Chloroflexi*, *Planctomycetes*, *Gemmatimonadetes* and *Nitrospirae*, but their relative abundances differed in each sample, resulting in different bacterial assemblages. The bacterial community from M2 and M3 sites showed no significant differences between each other (p = 0.9753). Nevertheless, they were significantly more diverse than M1 (p<0.001 and p<0.001, respectively) because sequences affiliated with *Proteobacteria*, *Firmicutes*, *Acidobacteria*, *Chloroflexi* and *Actinobacteria* covered more than 89% of the total classified bacterial sequences present in M1 but these phyla were present in a lesser proportion in M2 and M3 sites. The increase in diversity along the course of the river from east (M1) to west (M3) was associated with an increase in TOC and a decrease in As concentration. Concordantly, other authors described that moderately disturbed conditions, as a higher arsenic concentration in groundwater and sediment samples, often result in low diversity of microbial communities [56, 59], like those occurring at the origin of Camarones river (site M1). Gu et al. [60] reported that the structure of a bacterial community varied depending on the levels of arsenic contamination, showing an increase in the diversity of arsenic functional genes and 16S RNA genes as arsenic levels increased. Sheik et al. [61] had previously reported that arsenic affected the structure, abundance and diversity of microorganisms present in a community. Our work showed that communities of the sediment from Camarones river also varied depending arsenic concentration. Quéméneur et al. [62], studying arsenic pollution of water caused by old mines in France, determined that arsenic levels affected the structure of the bacteria possessing the *aioA* gene, increasing the number of copies of this gene in the most polluted places.

Previous studies on prokaryotic diversity in metal-contaminated sediments from eutrophic environments reported the presence of *Proteobacteria* as predominant phylum being *Beta-proteobacteria* the dominant subclass, followed by *Bacteroidetes*, mainly involved in the transformation of nutrients [59,63]. On the contrary, bacterial sequences recovered in the present study were mainly related to the *Deltaproteobacteria* subclass and *Firmicutes*, which could be attributed to the oligotrophic condition of the sediments collected at M1 and M2 sites of Camarones river [64].

Members of *Firmicutes*, mainly represented by *Bacillus* genus, were the most abundant in M1. To date, several *Bacillus* spp. have been isolated as dissimilatory As (V)-reducing bacteria, and *B*. *selenatarsenatis* was reported to be able to release As from contaminated soils [13]. A great diversity was found within *Deltaproteobacteria*, mainly represented by anaerobic members involved in the metal reduction, and particularly in respiring arsenate through a detoxification pathway encoded by *ars* operon [65]. Sulphate-reducing bacteria are key mediators of anaerobic carbon cycling in sediments and some genera, such as those related to *Anaeromyxobacter*, *Desulfobacca* and *Geothermobacter*, have been reported to play an important role in arsenate reduction [66].

High abundances of *Acidobacteria* and particularly those of GP6, retrieved across all samples, and of GP2, the most abundant in M2 site, may be related to their high metal resistance capability [56]. Sequences related to the genus *Caldimicrobium* (*Thermodesulfobacteria*), grouping extremely thermophilic, strictly anaerobic, sulphur-oxidizing bacteria, widely distributed in other hot environments [31], were dominant in the upper sites (M1 and M2) of Camarones river.

Differently to the other sites, genera identified in M3 were characteristically related to *Gammaproteobacteria*, and principally to *Ectothiorhodosinus*, an anoxygenic phototroph that may contribute to the primary production in halophilic conditions.

A total of 51 heterotrophic As-resistant strains able to survive under high arsenic concentrations, by means of genetic As resistance mechanisms, such as oxidation of As(III) or reduction of As(V), were isolated and identified as member of *Alpha*-, *Beta*- and *Gammaproteobacteria*, and *Firmicutes*. All arsenite resistant strains (17) possessing the *aio* gene were able to oxidize As(III) present in the culture medium into As(V) with values ranging from 68 to 100%. Particularly, arsenite oxidizing strains present at M1 showed the highest As(III) oxidizing percentages (90–100%), where the highest concentration of total arsenic was registered, confirming data previously reported in the same riverine zone [37, 38]. *Pseudomonas arsenicoxydans*, isolated from M1 and firstly described from sediment of the same area, represents and indigenous species in Camarones river with the highest ability to oxidize As (III)[37].

Costa et al. (2015) [67], isolated 123 arsenic resistant bacteria from sediments affected by gold mining activity and their 16S rRNA gene analyses showed the presence of 20 genera belonging to *Proteobacteria*, *Firmicutes* and *Actinobacteria*. They reported that the predominant gene was *arsC* (85%), which was also the predominant gene in our study (present in 50 of 51 strains), followed by *aioA* (20%), which accounted for 33% in our study. Escalante et al. (2008) [38] also reported that the major part of As-resistant strains isolated from Camarones river were able to the reduce arsenic. Overall, a significant correlation was observed between the environmental As distribution and the capacity to oxidize or reduce the metalloid present in the sediments of Camarones river.

The diversity of strains possessing *arsC* gene greatly increased as the total As concentration decreased in the river. As(V)-resistant strains isolated from M1 presenting the highest arsenic reducing capacity were identified as species of the following genera: *Pantoea* (98%), *Pseudomonas* (95%) *Sphingomonas* (80%), *Aeromonas* (75%) and *Acinetobacter* (70%), all of them that have been previously isolated from samples collected from the same riverine zone [36]. In contrast, *Fusibacter paucivorans*, isolated from M2, a thiosulfate-reducing bacterium, has not been described before as a As(V)-reducing bacterium.

Moreover, the co-presence of *aio* and *arsC* genes could confer higher resistance to arsenic than bearing *ars*C alone [21]. As-resistant strains possessing both genes in the sediments of Camarones river were: *Aquabacterium* sp., *Alcaligenes* sp., *Burkholderia cepacia*, *Pseudomonas marginalis*, *P*. *stutzeri*, *P*. *vancouverensis*, *P*. *putida*, *Xanthomonas* sp. and *Shewanella* sp. Of them, *Burkholderia cepacia* and *P*. *marginalis* were previously isolated from a natural biofilm associated to volcanic rocks of the Atacama Desert [36–38]. *Aquabacterium* sp., a well-known biofilm forming bacterium, *Bacillus* sp. AS-38 and *Xanthomonas* sp. are here reported for the first time as able to oxidize and also reduce arsenic. Moreover, this is the first report describing the presence of *arsC* gene and As(V)-reducing capability in *P*. *marginalis*, *P*. *stutzeri*, and *P*. *vancouverensis*.

Arsenate reduction and arsenite oxidation under aerobic conditions have been reported as detoxification mechanisms in several aerobic bacteria isolated from different As-contaminated sites [68, 69], suggesting that As (As[V]/As[III]) resistance plays an important role in the biogeochemical cycling of this element in nature [70]. Microbial species that bio-transform arsenic between oxidation states with differing environmental behaviours are able to control the release of adsorbed arsenic from sediments into water and therefore they may be potentially utilized for bioremediation purposes. Resistant strains able to transform As(III) into As(V) with a high rate of efficiency, such as *P*. *arsenicoxydans* strain VC-17, will be further investigated for its biotechnological potential, to detoxify waters from the Camarones river.

## Supporting information

S1 Table Relative abundances of bacterial phylogenetic group from sediment samples (M1, M2 and M3) collected from Camarones River. Dominant phylogenetic groups (≥1% of total classified sequences) common to sediments (M1, M2 and M3) are represented in bold.
(DOCX)

S2 Table Closest GenBank match to the relative sequences of the strains isolated from the three samples (M1, M2 and M3) collected from the Camarones river. (DOCX)
