# Supplementary material for: Effect of the natural arsenic gradient on the diversity and arsenic resistance of bacterial communities of the sediments of Camarones River (Atacama Desert, Chile)
Source: PLoS One. 2018 May 1;13(5):e0195080. doi: 10.1371/journal.pone.0195080 (PMC5929503; doi:10.1371/journal.pone.0195080)
Supplement: S1 Table — Dominant phylogenetic groups (≥1% of total classified sequences) common to sediments (M1, M2 and M3) are represented in bold. (DOCX) [file pone.0195080.s001.docx]

**Supporting Information**

**S1 Table.** Relative abundances of bacterial phylogenetic group from sediment samples (M1, M2 and M3) collected from Camarones river. Dominant phylogenetic groups (≥1% of total classified sequences) common to sediments (M1, M2 and M3) are represented in bold.

**
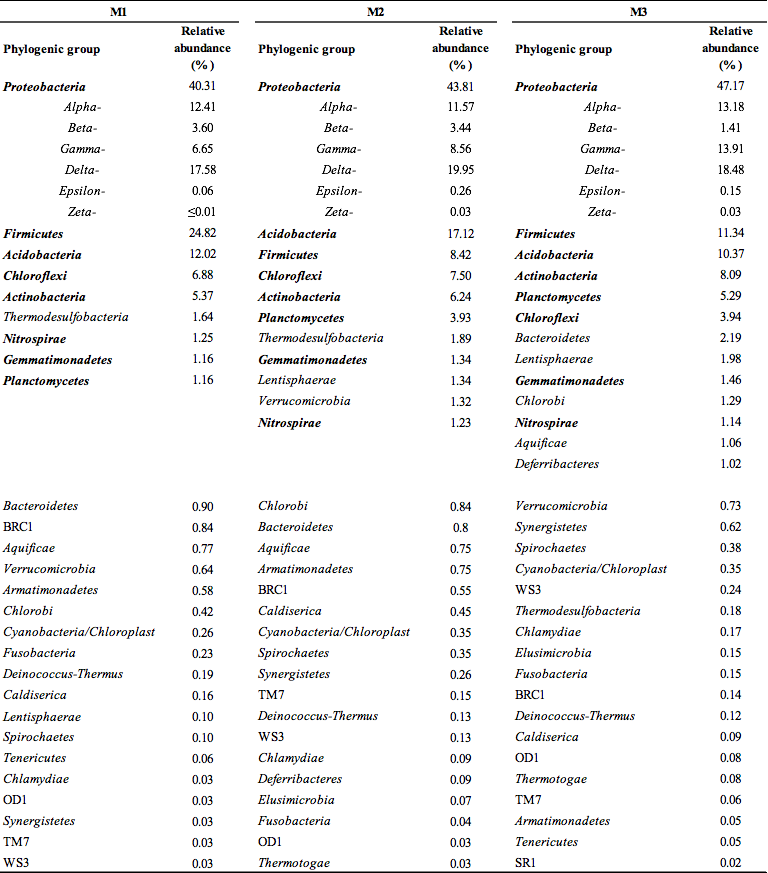
**
