# Supplementary material for: Effect of the natural arsenic gradient on the diversity and arsenic resistance of bacterial communities of the sediments of Camarones River (Atacama Desert, Chile)
Source: PLoS One. 2018 May 1;13(5):e0195080. doi: 10.1371/journal.pone.0195080 (PMC5929503; doi:10.1371/journal.pone.0195080)
Supplement: S2 Table — (DOCX) [file pone.0195080.s002.docx]

**S2 Table.** Closest GenBank match to the relative sequences of the strains isolated from the three samples (M1, M2 and M3) collected from the Camarones river.


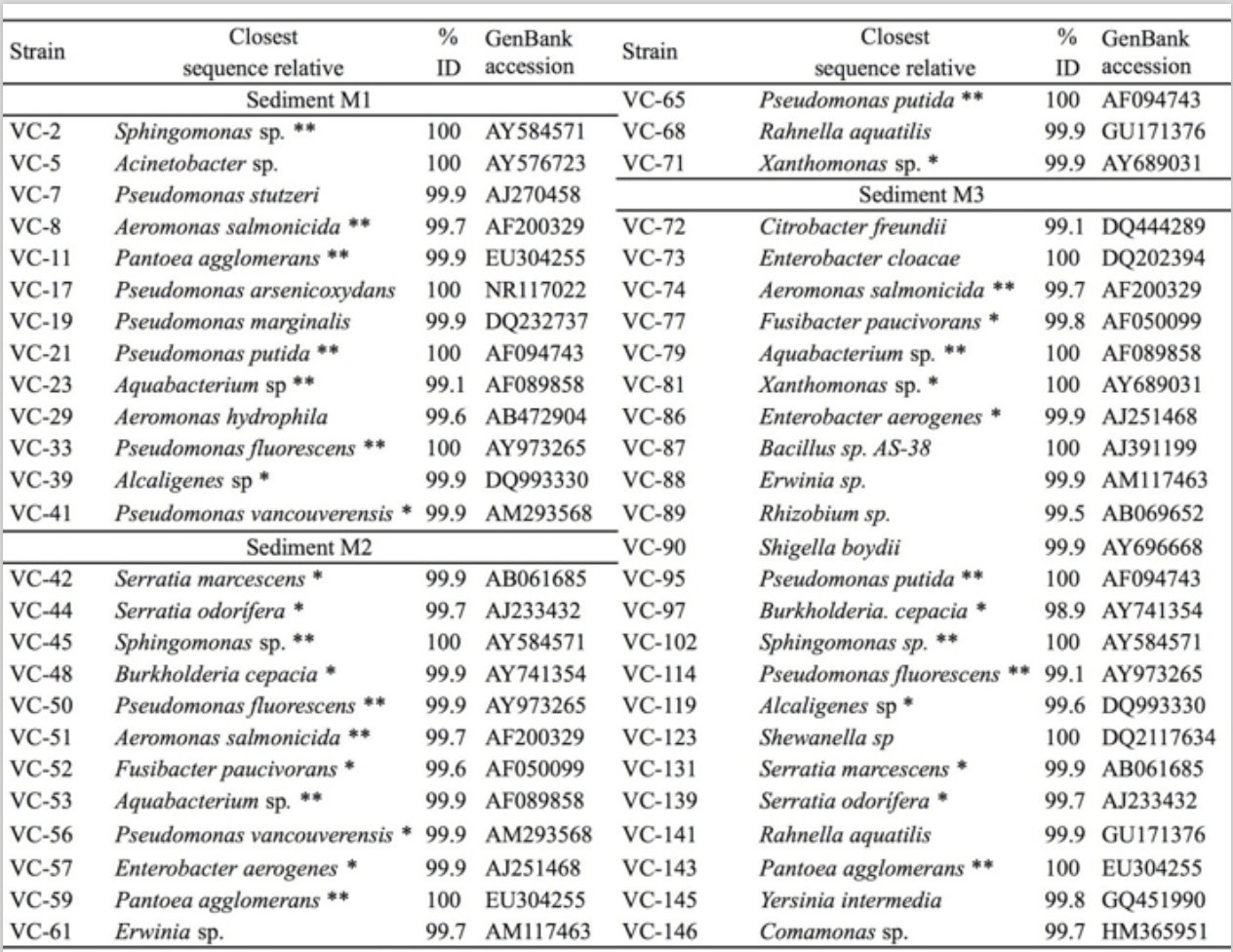


*Strain present in two sediment samples, ** Strain present in three sediment samples
